# Book Reviews

**Published:** 1894-11

**Authors:** 


### *BOOK REVIEWS.*

---

Diseases of the Chest, Throat, and Nasal Cavities, Including Physical Diagnosis and Diseases of the Lungs, Heart, and Aorta, Laryngology, and Diseases of the Pharynx, Larynx, Nose, Thyroid Gland, and Esophagus. By E. Fletcher Ingalls, A.M., M.D., Chicago. Published by Wm. Wood & Co., New York.

The third edition of this most excellent work of Dr. Ingalls has just been issued, and coming as it does within a year after the appearance of the second edition, it shows with what favor and success it has been received by the medical profession. This work is peculiarly acceptable, in that it treats of the whole respiratory system and each individual part which aids in its formation, taking into consideration at the same time the diseases of the circulatory system which is so closely related to and dependent upon the workings of the respiratory organs. Few men are so well qualified as Dr. Ingalls to combine a work on the diseases of the nose, throat, and larynx with that of the lungs and heart, for besides being an eminent professor of the practice of medicine in the Rush Medical College of Chicago, Dr. Ingalls is also a distinguished specialist on the nose and throat. Hence his peculiar fitness for the production of such a work as is now before us. While the work is by no means exhaustive, yet it is produced in a manner practical, concise, and easily understood, which is the *sine qua non* of any text-book. In the beginning of the preface to the third edition we find the real intent of the author, and as such the work will find a permanent place among American text-books. He says "This is not meant for an encyclopedic work but is intended to present in convenient form the known facts relating to diseases of the respiratory tract and circulatory organs, and I have brought their consideration under one cover because the parts are so closely related that, when one is diseased, it is generally necessary to interrogate the others before a correct diagnosis or proper plan of treatment can be reached." The first fifteen chapters treat of the diseases of the lungs and heart; from chapter sixteen to chapter twenty, inclusive, the author treats of the throat—including the larynx, pharynx, tonsils, etc.; from chapter twenty to twenty-six, inclusive, the nasal cavity is treated, while the last remaining chapter is devoted to diseases of the esophagus. Throughout the book the author is especially clear and explicit in the differential diagnosis of similar diseases, using for this purpose parallel columns in order to impress upon the reader these differential signs. We consider this feature of the book an especial mark of value. The illustrations and cuts are timely, and altogether the work is one which is destined to hold a firm place among the text-books on this subject.

R.

---

HOME TREATMENT FOR CATARRH AND COLDS. By Leonard O. Dessar, M.D., of New York. Published by Home Series Publishing Company, of New York.

In this day of quackery, where patent and proprietary medicines are endeavoring to supplant the rational use of remedies, it is not surprising that such a book as the above should be launched upon the public, in order to teach them a "short method of becoming physicians at home." The only surprise and regret to us, however, is that the author, who is a most distinguished and reputable physician, should allow his literary talents to be used in such a direction, and in this way countenancing the correctness of the principle. When "home-treatment books" upon the different diseases to which man is heir shall have been written and recommended by reputable physicians as guides to every household, then physicians must seek some other avocation, for their sphere of usefulness will then be limited and their means of livelihood eradicated forever. The principle is wrong, and still more in error is the practice. We do not believe in the need of such "household books," especially where instructions are given to use the medicines themselves without the advice of physicians, for every family physician, or in fact, every specialist, should so instruct their patients as to the proper hygienic care of their person and household, as to preclude the necessity of such books.

Essentials of the Diseases of the Ear. By E. B. Gleason, M.D., of Philadelphia. Published by W. B. Saunders, Philadelphia.

This little work is a late production from the well known publishing house of W. B. Saunders, being another one of the series of question compends, and, like all the others, is a most admirable work of its kind, and eminently meets the requirements for which it was intended. These compends are all written by competent men, and such as know the exact needs of students and practitioners. Dr. Gleason has already been introduced to us before as the joint author of the compend on "Essentials of Diseases of Eye, Nose, and Throat," and together with the present edition on the ear, the two will make a most admirable series for the students of medecine. It would have been much better, however, had the division been made so as to combine the Diseases of the Ear, Nose, and Throat, and give to Diseases of the Eye a separate compend of its own. This would have been much better, clinically—much more advantageous as reference books for students, since such a combination is now universally recognized as the best.

---

Materia Medica, Pharmacology, and Therapeutics. By W. Hale White, M.D., Lecturer on Materia Medica and Therapeutics at Guy's Hospital, London, etc. Edited by R. W. Wilcox, M.D., Professor of Clinical Medicine and Therapeutics in the New York Post Graduate Medical School and Hospital, etc. Second Edition. Revised. P. Blakiston, Son & Co., 1012 Walnut Street, Philadelphia.

This book will be found of special use by students of medicine. It is too condensed as a work of reference for the practitioner. The work bears some resemblance to that of Potter, but is not so complete. The classification of drugs is nearly the same. Not so much prominence is given to therapeutics. The work is thoroughly up to date, and contains conservative descriptions of the newest remedial agents whose places in therapeutics are not yet established. The classification of drugs is made from their physiological actions, probably the best arrangement, though not so convenient. We are glad to note the omission of certain preparations which have been about discarded both by medicine and pharmacy. Medical students will find this book exceedingly useful and practical.

NURSING IN PELVIC SURGERY. By L. S. McMurtry, M.D., Professor of Gynecology in the Hospital College of Medicine, Louisville. Published by John P. Morton & Co., Louisville, Ky.

This manual of ninety-two pages contains excellent directions upon the most important aspects of nursing in pelvic surgical cases. It is written by a practical surgeon, and is the expression of knowledge the result of experience. The chapters relate to the preliminary preparation of the patient for the operation, the preparation of instruments, sponges, and dressings, the conduct of the operation, the after-attention, etc. The book is simply, but carefully written, representing accurately the present system of nursing in abdominal surgery. The principles and technique herein described should be familiar to every one who has any relation with this sort of work, from nurse to operating surgeon. Certain complications and unpleasant results might thus be prevented, which sometimes come unwished for.

---

LIPPINCOTT'S MAGAZINE FOR NOVEMBER, 1894.

The complete novel in the November issue of *Lippincott's* is "Dora's Defiance," by Lady Lindsay, an author who has made her mark in England, though little known as yet in this country. It is a brightly told story of a very peculiar young lady who could find no interest in life till it came too late to be taken in the conventional way.

"An Arizona Speculation," by Mary E. Stickney, has the full Western flavor, and depicts a character evidently drawn from life. Ella Higginson narrates briefly but forcibly a tragical episode, "In the Bitter Root Mountains." In "Rector Warne's Heresy," Gillam W. Ford shows how duty came to the front and drove doctrine into the background. Virginia Woodward Cloud brings to life "The Man who died at Amdheran," and gives him something to live for. "The Roses" of which Fannie E. Newberry tells were sent to the wrong lady, with curious results.

Under the heading, "Ten Dollars a Day—No Canvassing," Philip G. Hubert, Jr., discusses some queer circulars and the dubious opportunities of wealth they offer. W. S. Walsh collects a number of interesting anecdotes of dignitaries and others who have gone about "Incognito." E. J. Gibson explains the labors of "The Washington Correspondent," and Frederic M. Bird discourses on "Magazine Fiction, and How Not to Write It."

Passing to distant lands, we go "Bargaining in Russia" with Isabel F. Hapgood, and learn about "Rabbits in New Zealand" from J. N. Ingram. Coming home again, we listen to Colonel Richard Malcolm Johnston's recollections of "My Schools," and to Edgar Fawcett's of "Old New York Restaurants."

